# Space maintenance in autogenous fresh demineralized tooth blocks with platelet-rich plasma for maxillary sinus bone formation: a prospective study

**DOI:** 10.1186/s40064-016-1886-1

**Published:** 2016-03-07

**Authors:** Eun-Suk Kim, Ji-Yeon Kang, Jae-Jin Kim, Kyoung-Won Kim, Eun-Young Lee

**Affiliations:** Department of Oral and Maxillofacial Surgery, Weerae Dental Clinics, Seoul, Korea; Department of Oral and Maxillofacial Surgery, Dongtan Sacred Heart Hospital, Hallym University, Hwaseong, South Korea; Department of Oral and Maxillofacial Surgery, Chungnam National University School of Medicine, Daejeon, Korea; Department of Oral and Maxillofacial Surgery, College of Medicine and Medical Research Institute, Chungbuk National University, Chungbdaero 1, Seowon-gu, Cheongju, Chungbuk 28644 South Korea

**Keywords:** Autogenous fresh demineralized tooth, Block, Platelet-rich plasma, Allograft, Xenograft, Powder

## Abstract

This prospective study evaluated the effectiveness of autogenous fresh demineralized tooth block (Auto-FDT block) with platelet-rich plasma (PRP) for maxillary sinus augmentation with simultaneous implant installation. Auto-FDT block with PRP was used in Group 1 (n = 15) and combined graft (allograft and xenograft) powder with PRP was used in Group 2 (n = 15). For up to 2 years after the final prosthesis was installed, clinical and radiographic examinations were performed to evaluate the amount of graft materials, residual alveolar height (RAH), sinus height (SH) after grafting, augmented graft height (AGH) and resorption height (RH). In ten cases, biopsies were harvested for histological and histomorphometric analyses. A total of 59 implants were placed in a severe atrophic posterior maxilla with less than 5 mm of RAH and sinus augmentation. None of the patients developed sinusitis or other complications, such as implant loss. The graft material extracted included one molar (or 2 premolars) in Group 1 and 1.8 cc in Group 2. The radiologic examination revealed the following average between-group difference SH (Group 1, 14.12 ± 1.63 mm vs Group 2, 16.51 ± 1.29 mm) and AGH (Group 1, 11.62 ± 2.22 mm vs Group 2, 13.65 ± 1.35 mm). However, sufficient SH and AGH were observed for the implants in the Auto-FDT block group. Two years after final prosthesis was installed, no between-group difference in the RH was observed (Group 1, 1.23 ± 0.73 mm vs Group 2, 1.77 ± 0.54 mm, *P* = 0.021). The histomorphometric analysis revealed no between-group difference in the new bone volume (Group 1, 23.13 ± 1.42 % vs Group 2, 24.18 ± 2.19 %, *P* = 0.548). The results showed that Auto-FDT block with PRP can be used in grafted sinuses for implants with only one extracted molar (or two premolars). Auto-FDT block with PRP promotes new bone formation that is comparable with combined grafts. Auto-FDT block with PRP is as an alternative to bone grafting and can be a predictable procedure for sinus augmentation.

## Background

Maxillary posterior tooth loss leads to bone loss in the maxillary sinus floor and increases pneumatization of the maxillary sinus (Hatano et al. 2004). The placement and integration of implants in the maxillary posterior edentulous area requires sinus floor augmentation. The traditional technique for sinus floor augmentation is the lateral window technique which involves filling with a powdered bone substitute (Boyne and James 1980). Various bone substitutes have been used to fill the space of the maxillary sinus, including autogenous bone, allograft, xenograft, synthetics, demineralized autogenous teeth and a combination of various materials (Klijn et al. 2010; Schlegel et al. 2003; Wheeler 1997; Wiltfang et al. 2003; Kim et al. 2014; Lee and Kim 2009).

However, there are still unresolved problems related to graft materials, such as unpredictable resorption, limited amounts and required donor sites of autologous bone, the inability of alloplastic materials to form bone, and the slow resorption ability of xenograft materials (Hatano et al. 2004; Schlegel et al. 2003; Kim et al. 2014; Iezzi et al. 2012). In addition, there is a need for a large amount of sinus filling. An average of 5–6 cc of autogenous bone is required for one sinus. For a combination graft, an average of 2–3 cc of bone is required (Peleg et al. 2004). To solve these problems, some studies have demonstrated bone formation using the patient’s own blood or platelet-rich plasma (PRP) to fill void without bone grafts (Chen et al. 2007; Mazor et al. 2004; Moon et al. 2011). Although previous studies have reported good bone formation without bone graft, few studies have described the long-term follow-up of bone graft cases (Moon et al. 2011; Sohn et al. 2011). The augmented height and area are significantly decreased in the sinus augmentation using blood clot without bone grafts.

We focused on demineralized tooth block graft to solve the problems associated with previous sinus bone grafts. Recently, the use of tooth grafts has increased in alveolar bone and sinus defects. Demineralized autogenous teeth can provide effective graft material (Kim et al. 2014; Yeomans and Urist 1967). Demineralized autogenous tooth graft materials were made from discarded teeth, such as freshly extracted 3rd molars, and periodontic and/or endodontic problem teeth. In previous experimental studies, human teeth have been proven to be biocompatible materials that demonstrate both osteoinductive and osteoconductive properties (Yeomans and Urist 1967). However, the disadvantage of tooth graft material is the limited amount available for sinus graft. To solve this problem, we used a block rather than a powder with PRP for space maintenance.

This prospective study evaluated the use of autogenous fresh demineralized tooth block (Auto-FDT block) with PRP compared with a combined graft (allograft and xenograft powder) for maxillary sinus augmentation with a residual alveolar height (RAH) of less than 5 mm.

## Results

### Clinical and radiologic observations

All patients were followed for 2 years after the final prosthesis implant. None of the patients dropped out, and no complications were observed during the surgical procedures. Group 1 consisted of 15 patients (10 males and 5 females; mean age 54.9 years; range 39–68 years) who underwent sinus augmentation using Auto-FDT block. Group 2 consisted of 15 patients (9 males and 6 females; mean age 54.3 years; range 41–68 years) who underwent sinus augmentation using allograft and xenograft powder. In Group 1, 28 implants were placed in the premolar and/or molar areas. In Group 2, 31 implants were placed in the premolar and/or molar areas.

**Fig. 1:**
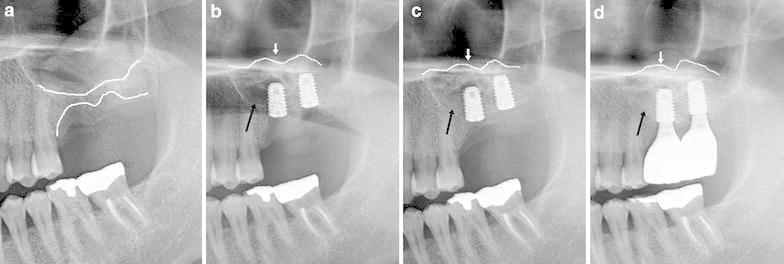
Panoramic radiography of Auto-FDT block with PRP. **a** Preoperative panoramic view showing severe atrophy in the Lt. maxillary posterior area. **b** Immediate postoperative panoramic view. Image showing implants placement and Auto-FDT block graft in the sinus area and empty space (radiolucent space) between the Auto-FDT block and residual alveolar bone (*black arrow*). **c** The graft (after 6 months) showing increased radiopaque in the sinus area (*black arrow*). **d** After 2 years, there was greater radiopacity in the grafted sinus area using block Auto-FDT and PRP [the final prosthesis without resorption compared with the panoramic view during the uncovering surgery (**c**, *black arrow*)]. The implants were successful and were fully surrounded by bone. Residual alveolar bone (*white solid line*) and sinus graft height (*white dotted line* and *white arrow*)

All patients healed uneventfully, and no signs or symptoms of maxillary sinus disease were observed. The mean Implant Stability Quotient (ISQ) of the implants was 64.2 ± 5.2 (55–81) in Group 1 and 69.4 ± 6.2 (59–83) in Group 2. There was no significant difference between the groups (*P* = 0.162). No implant failure (in either group) was observed during the follow-up period. After an average of 6 months was allowed for the implants to integrate, new bone formation in the maxillary sinus was observed on the radiographs. Follow-up panoramic radiography showed good bone formation in both groups. In particular, the empty space among the block of the Auto FDT filled in PRP gradually exhibited new bone formation (Fig. 1a–d).

Tables 1 and 2 summarizes the implant sites, extracted teeth for the Auto-FDT block, amount of graft materials and measuring heights, such as RAH, SH1, SH2, AGH and RH for both groups. The RAH ranged from 1.20 to 4.20 mm in Group 1 and from 1.55 to 3.85 mm in Group 2. There was no significant between-group difference in the RAH (*P* = 0.233). The mean SH1 was 14.12 ± 1.63 mm (Group 1) and 16.51 ± 1.29 mm (Group 2) for the immediate graft. The mean SH2 was 12.89 ± 1.07 mm and 14.75 ± 1.31 mm after 2 years the final prosthesis. In Group 1, the amount of graft materials from one molar (or 2 premolars) was used, and the AGH averaged 11.62 ± 2.22 mm. In Group 2, the AGH averaged 13.65 ± 1.35 mm using 1.8 cc of graft material. The radiologic examination indicated significant between-group differences in SH and AGH. In the SH1 comparison between the two groups, Group 1 was approximately 2.3 mm less compared with Group 2. SH2 was similar to SH1. However, it was possible to support the implants and adequate sinus graft height with the Auto-FDT block graft. In addition, there was no significant between-group difference in the decreased height during the follow-up period (RH; 1.23 ± 0.73 mm in Group 1 vs 1.77 ± 0.54 mm in Group 2, *P* = 0.021) (Table 3).

**Table 1:** The installed implant site, grafted material amount, and height measurement in Group 1

No.	Implant sites	Amount of Auto-FDT block (extracted tooth)	IL (mm)	RAH (mm)	SH1 (mm)	AGH (mm)	SH2 (mm)	RH (mm)
1	#15, 16	#18	8.5,11.5	3.41	11.85	8.44	11.70	0.15
2	#26, 27	#16	8.5, 8.5	1.30	13.45	12.15	12.47	0.98
3	#16, 17	#48	8.5, 8.5	2.40	12.20	9.80	11.25	0.10
4	#16	#37	10	3.50	11.90	8.40	11.80	0.95
5	#16, 17	#37	10, 10	1.65	15.50	13.85	13.60	1.90
6	#27	#27	8.5	1.80	12.90	11.10	12.30	0.60
7	#26, 27	#17	8.5, 10	2.80	14.65	11.85	13.70	0.95
8	#26, 27	#48	10, 10	1.70	16.35	14.65	13.45	2.90
9	#25, 26	#18	10, 10	1.55	16.95	15.40	15.35	1.60
10	#16, 17	#17	8.5, 8.5	1.70	14.85	13.15	13.35	1.50
11	#15,16, 17	#48	10, 10, 10	3.47	15.23	11.76	13.60	1.63
12	#15, 16	#38	10, 10	4.20	12.60	8.40	12.00	0.60
13	#26, 27	#24, 25	8.5, 10	3.00	13.60	10.60	12.15	1.45
14	#25, 26	#48	10,11.5	1.20	14.30	13.10	13.00	1.30
15	#17	#46	8.5	3.80	15.50	11.70	13.70	1.80

*IL* implant length, *RAH* residual alveolar height, *SH1* sinus height at immediate graft, *AGH* augmented graft height (SH1 − RAH), *SH2* sinus height after 2 years the final prosthesis, *RA* resorption height of the graft

**Table 2:** The installed implant site, grafted material amount, and height measurement in Group 2

No.	Implant sites	Material amount: A and X (cc)	IL (mm)	RAH (mm)	SH1 (mm)	AGH (mm)	SH2 (mm)	RH (mm)
1	#16, 17	1.8	10, 8.5	3.55	17.65	14.10	16.05	1.60
2	#25, 26, 27	1.8	8.5, 8.5, 8.5	3.53	16.17	12.64	13.97	2.20
3	#16	1.8	11.5	1.55	17.35	15.80	15.35	2.00
4	#15	1.8	11.5	2.90	16.90	14.00	15.90	1.00
5	#16, 17	1.8	11.5, 10	2.75	18.05	15.30	15.95	2.10
6	#15, 16	1.8	11.5, 10	3.85	16.10	12.25	14.95	1.15
7	#15, 16	1.8	11.5, 10	3.65	16.80	13.15	15.75	1.05
8	#26, 27	1.8	11.5, 8.5	2.25	15.90	13.65	13.85	2.05
9	#15, 16	1.8	11.5, 8.5	2.85	16.10	13.25	14.20	1.90
10	#14, 15, 16	1.8	11.5, 11.5, 10	2.77	18.67	15.90	17.07	1.60
11	#25, 26, 27	1.8	11.5, 10, 10	3.00	17.63	14.63	15.23	2.40
12	#16, 17	1.8	8.5, 8.5	1.85	14.65	12.80	13.40	1.25
13	#26, 27	1.8	11.5, 10	3.10	16.75	13.65	14.15	2.60
14	#26, 27	1.8	8.5, 8.5	1.75	14.10	12.35	12.90	1.20
15	#16, 17	1.8	8.5, 8.5	3.70	14.90	11.20	12.50	2.40

Bio-Oss are packed in grams and were measured by volume (cc)

*IL* implant length, *RAH* residual alveolar height, *SH1* sinus height at immediate graft, *AGH* augmented graft height (SH1 − RAH), *SH2* sinus height after 2 years (the final prosthesis), *RA* resorption height of the graft, *A* allograft, *X* xenograft

**Table 3:** Mann–Whitney U test of measured heights between groups

	Auto-FDT new bone (Group 1, n = 15)	Allograft and xenograft, new bone (Group 2, n = 15)	*p* value
	Mean ± SD (mm)	Mean ± SD (mm)	
RAH	2.50 ± 1.01	2.87 ± 0.74	0.233
SH1	14.21 ± 1.63	16.51 ± 1.29	0.000
SH2	12.89 ± 1.07	14.75 ± 1.31	0.000
AGH	11.62 ± 2.22	13.65 ± 1.35	0.007
RH	1.23 ± 0.73	1.77 ± 0.54	0.021

*RAH* residual alveolar height, *SH1* sinus height at immediate graft, *AGH* augmented graft height (SH1 − RAH), *SH2* sinus height after 2 years (the final prosthesis), *RA* resorption height of the graft

### Histological and histomorphometric results

**Fig. 2:**
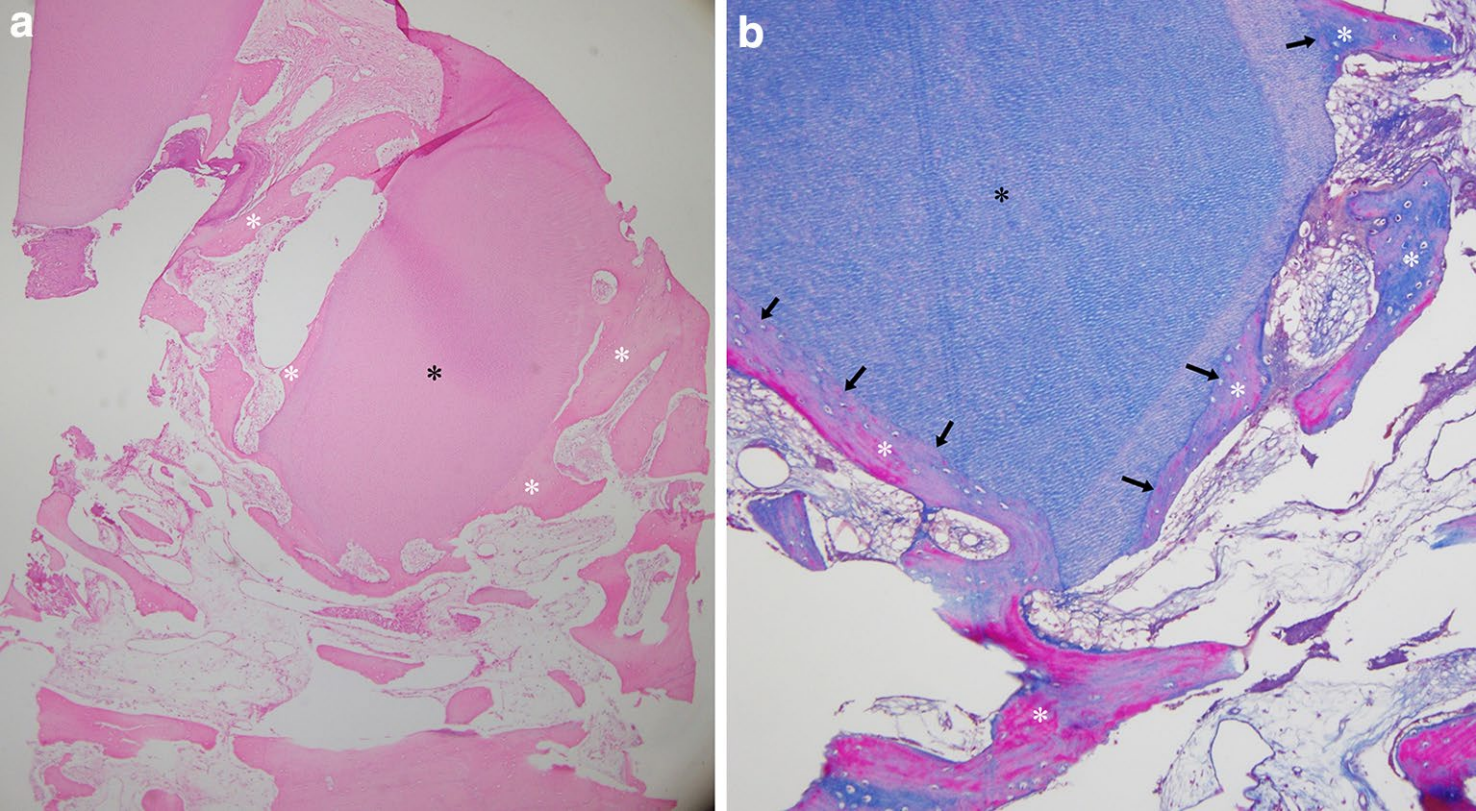
Histologic features of specimen (6 months after grafting). **a** The graph showed good to excellent new bone formation with dense connective tissue in the empty space between the Auto-FDTs. (H&E, ×100). **b** New bone connected with the border of Auto-FDT. (Masson’s trichrome staining, ×200). The Auto-FDT block (*black asterisk*), new bone (*white asterisk*), and border between the Auto-FDT and new bone (*black arrow*)

In all groups at low magnification, resorbable graft materials and newly formed bone were observed. Newly formed bone tissue was observed in the empty space between the Auto-FDT bocks with resorbed Auto-FDT. Osteocytes were found to be regularly and evenly dispersed in the new bone area in hematoxylin-eosin (H&E) stained sections, which showed healthy bone (Fig. 2a). A Masson’s trichrome (MT) stained section demonstrated direct integration between newly formed bone and Auto-FDT (Fig. 2b).

The most intact biopsies were chosen for the histomorphometry. For both groups, 10 slides were used for the histomorphometric analysis. The measurements were performed at 100× magnification above the residual alveolar bone. The portion of new bone formation was 23.13 ± 1.42 % in Group 1 and 24.18 ± 2.19 % in Group 2 (Table 4). There were no significant between-group differences (*P* = 0.548). The area of residual graft material measured 22.21 ± 1.19 % (Group 1) and 31.18 ± 2.09 % (Group 2). There was a significant difference in the residual graft material (*P* = 0.008). The difference was between the block and powder. More space was observed between the blocks compared with the powders in the measured area.

**Table 4:** Mann–Whitney U test of measured new bone and residual graft material between groups

	Auto-FDT new bone (Group 1, n = 5)	Allograft and xenograft new bone (Group 2, n = 5)	*p*-value
	Mean ± SD	Mean ± SD	
New bone			
Area (mm^2^)	3.41 ± 0.21	3.57 ± 0.32	0.548
Percent	23.13 ± 1.42	24.18 ± 2.19	
Residual graft material			
Area (mm^2^)	3.28 ± 0.18	4.60 ± 0.31	0.008
Percent	22.21 ± 1.19	31.18 ± 2.09	

*RAH* residual alveolar height, *SH1* sinus height at immediate graft, *AGH* augmented graft height (SH1 − RAH), *SH2* sinus height after 2 years the final prosthesis, *RA* resorption height of the graft

## Discussion

It is important to provide sinus bone augmentation for implant installation to obtain volumetric stability of the grafted sinus, to minimize donor site morbidity, to minimize the patient’s and clinician’s perceived complexity of the surgery, and to minimize costs and operation times. A conventional technique for sinus augmentation is the lateral approach using powders from various bone graft materials. It is a relatively simple and predictable surgical technique (Chen et al. 2007). This approach permits the surgeon to gain direct access to the sinus floor. However, it requires a significantly large volume of graft material and results in high costs and donor site morbidity for sinus augmentation (Peleg et al. 2004; Chen et al. 2007).

Some studies have reported the success of implants exclusively using elevation of the Schneiderian membrane, peripheral blood and PRP without any graft material. In particular, PRP is an important source of multiple growth factors to increase the rate of bone formation in a graft (Mazor et al. 2004; Moon et al. 2011). Good bone regeneration has been obtained with blood clots or PRP alone without additional bone graft materials. Moon et al. reported new bone formation following the application of peripheral venous blood in the space between the elevated sinus membrane and simultaneously placed implants; the overall implant survival rate was 93.5 % (Moon et al. 2011). However, the residual alveolar heights of most cases (n = 26) were above 4 mm; two of five cases with less than 4 mm of residual alveolar height were determined to be failures. In most cases (n = 19), the sinus grafted heights decreased to less than or equal to the implant length during the follow-up period (2 years). Pinchasov et al. reviewed the scientific literature regarding bone formation in the sinus after the membrane elevation procedure without using any bony substitutes from 1993 to 2013 (Pinchasov and Juodzbalys 2014). A total of 19 studies were included in this review. This study showed that the residual alveolar height was more than the average of 5 mm, and the bone gain ranged from 4.5 to 8.2 mm. Furthermore, to improve the amount of new bone formation, space-maintaining devices were used, such as hollow hydroxylapatite space-maintaining devices and titanium bone fixation devices.

Xu et al. showed that most of the newly formed bone in the augmented sinus space disappeared 10 weeks after blood-clot implantation without bone graft materials (Xu et al. 2005). The augmented height significantly decreased from 2 to 10 weeks. This finding is related to the enlargement of the sinus at the expense of the alveolus and bone resorption resulting from the increased osteoclastic activity of the periosteum of the Schneiderian membrane and increased pneumatization of the sinus simply because of the increase in positive sinus air pressure. The authors suggested that the blood clots might not withstand the sinus air pressure, and some materials, such as freeze-dried demineralized bone and autogenous bone, cannot withstand sinus pressure during the first several weeks and lose density and height over time.

To prevent resorption of the grafted material due to sinus air pressure, xenograft was used in the sinus augmentation. The xenograft particles provided an ideal scaffold for osteoconduction. For a certain period after the implantation, the augmented height was virtually unchanged (Hatano et al. 2004; Xu et al. 2004). These findings indicated that the particles inhibited pressure-induced osteoclastic bone resorption in the sinus spaces segmented by the xenografts (Xu et al. 2004). However, the xenograft appears to undergo slow or even no resorption for up to 6 years, as confirmed by clinical biopsies (Schlegel and Donath 1998; Hallman et al. 2001). Non-resorbable graft materials will not remodel and functionally adapt to the surrounding bone.

This study was performed as an alternative to solve the disadvantages of these methods. We attempted to use autogenous extracted tooth block for sinus augmentation. While the extracted tooth is an autogenous graft, there was no donor site morbidity. Using the block technique, it was possible to fill a sinus with a small amount of bone substitutes and to perform a simple surgical procedure compared with the powders (Fig. 4). The graft height using the allograft and xenograft powder (amounts; 1.8 cc) was higher than the graft height using the Auto-FDT block (one molar) (*P* = 0.007). Although there was a significant difference, the grafted sinus floor was above the implant apex in Group 1 for 2.5 years during the follow-up period. Auto-FDT block requires only one molar for one sinus augmentation and also minimizes patient costs because the extracted tooth is used. The height of the grafted bone decreased during the first 2–3 years after the sinus augmentation (Hatano et al. 2004). The difference in the resorbed height (in both groups) was not statistically significant during the first 2.5 years after the sinus augmentation. The resorption height of Group 1 (1.23 ± 0.73) was less than Group 2 (1.77 ± 0.54). Our data showed that the Auto-FDT block contributed to the maintenance of space to prevent sinus air pressure. In the clinical and radiographic analyses of this study, Group 1 showed a 100 % implant success rate at the end of the study.

Jun et al. reported that there was no significant difference in the portions of new bone formation and residual graft material between the autogenous tooth powder group and the Bio-Oss powder group (Jun et al. 2014). In our study, there was no statistically significant difference in the new bone formation between the two groups. However, the portion of residual graft material was smaller in Group 1 (22.21 ± 1.19) compared with Group 2 (31.18 ± 2.09). The empty spaces of the Auto-FDT block were larger than the composite powder graft. Bone formation was observed in the empty space in the Auto-FDT block and remodeling (Fig. 1d).

The characteristics of a bone substitute for sinus augmentation can be used as a scaffold, with space maintenance. Adequate pore volume is required for vascular invasion, and the regenerated tissue exhibited mechanical characteristics (Iezzi et al. 2012). An ideal grafting material in sinus augmentation provides biologic stability, ensures volume maintenance and induces the formation of vital bone and bone remodeling for implants while preventing pneumatization. Auto-FDT block for sinus augmentation provided space maintenance to prevent repneumatization. Additionally, some authors have reported that autogenous demineralized teeth stimulated bone formation (Kim et al. 2014; Yeomans and Urist 1967; Lee et al. 2014). Demineralized tooth is rich in growth factors, such as bone morphogenetic proteins (BMP), transforming growth factor-beta (TGF-beta), fibroblast growth factor (FGF), platelet-derived growth factor (PDGF) and epidermal growth factor (EGF) (Urist et al. 1984; Gomes et al. 2006). At six postoperative months, histologic sections featured the remodeling phase of new bone in the space between Auto-FDT blocks (Fig. 2a). Signs of resorption were observed in the borders of Auto-FDT blocks. MT stained sections revealed that their integration was as tight as fusion or interdigitation (Fig. 2b). Demineralized teeth exhibit osteoconductive and osteopromotive properties, which are incorporated into the bone repair process and can induce bone formation using bone regeneration techniques (Gomes et al. 2006). To promote bone formation and fill the void between the Auto-FDT blocks, autologous PRP was applied in this study. PRP can be added to the graft matrix to enhance the growth factors. PRP used a volume of autologous plasma with a platelet concentration greater than baseline (at least 1000,000 platelets/µl according to the manufacturer’s instructions). Using the radiographic view, we observed new bone formation (radiopaque image) in the PRP space under the Auto-FDT block (Fig. 1d).

## Conclusions

Auto-FDT was recycled for use as a graft material. Using only a small amount of Auto-FDT block (one extracted tooth) and PRP allowed successful sinus bone grafts without donor site morbidity or commercial graft materials, which resulted in cost savings for both the patient and the physician. In the future, more studies will be needed to evaluate Auto-FDT block applications for other bone graft procedures. This study clearly showed that implant placement using Auto-FDT block with PRP can be a predictable procedure for sinus augmentation with simultaneous implant installation.

## Methods

### Study subjects

This study was approved by the Institutional Review Board at Chungbuk National University Hospital, Cheongju, Chungbuk, Korea (IRB Number: D2011-02-002), and the guidelines for good clinical practice were respected. All patients provided informed consent. Thirty healthy adult patients with non-contributory past medical histories were included in this study. The following inclusion criteria were applied: patients who were partially edentulous in maxillary premolar or molar areas with severe alveolar atrophy and less than 5 mm of RAH. The exclusion criteria were as follows: any pathologic maxillary sinus condition, uncontrolled periodontitis, unrepaired sinus membrane perforation, heavy smokers, head and neck radiation therapy, chemotherapy and loss of initial implant stability. Using a random sequence generator, the patients were divided into two groups. The experimental group consisted of 15 patients (Group 1) who had undergone sinus augmentation using Auto-FDT block with PRP. The control group consisted of 15 patients (Group 2) who had undergone sinus augmentation using allograft (SureOSS, 850-1000 μM, Hansbiomed, Seoul, Korea) and bovine xenograft (Bio-Oss^®^ 0.25–1 mm, Geistlich Biomaterials GmbH, Baden–Baden, Germany) with PRP. Preoperatively, the patients were informed of the surgical procedures. Before the sinus grafting and implant placement, the patients underwent oral and physical examinations, and conventional panoramic radiography (Pax-i3D, Vatec, Seoul, Korea) was performed.

### Auto-FDT block and PRP preparation for Group 1

**Fig. 3:**
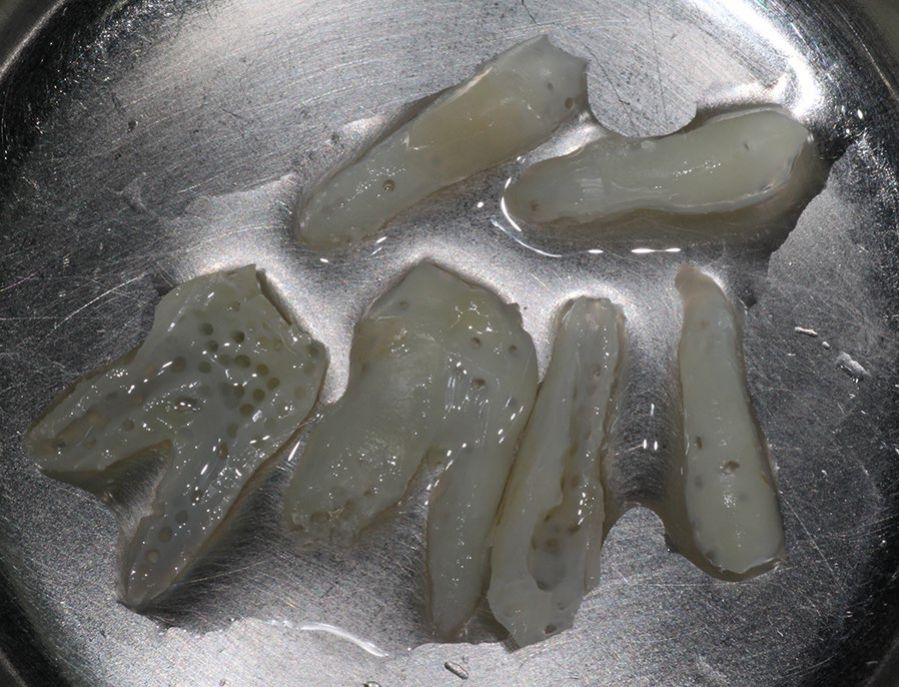
Preparation of Auto-FDT block, one molar and two premolars were prepared

The teeth that required extraction exhibited advanced periodontal or periapical disease or non-restorable decay or were non-functional third molars. Remnant tissues around the root of the extracted tooth were removed using a surgical blade or periosteal elevator. Using a pear-shaped carbide bur (N. 330), pulp tissue from the pulp chamber was removed, and many small holes were made on the surface of the tooth to effectively perfuse the reagent (Fig. 3). The trimmed tooth was washed in the processing reagents (i.e., superoxide, ethanol and distilled water) and frozen at −20 °C until the grafting surgery. Demineralization, washing, and sterilization of Auto-FDT were performed in an ultrasonic chamber equipped with vacuum and cooling units (VacuaSonic™, CosmoBioMedicare, Seoul, Korea) on the day of surgery or the day before surgery according to the manufacturer’s instructions. Periodic negative pressure was applied during low frequency ultrasonic treatment at a temperature below 40 °C. Each reagent used for the tooth treatment was kept in a sterile bioreactor tube with a 0.2 μm pore size polytetrafluoroethylene (PTFE) filter. The whole preparation process required 120 min. The Auto-FDT block was placed into a storage container and kept refrigerated or frozen without freeze-drying. The Auto-FDT block was provided in four pieces for one sinus augmentation surgery.

All PRP were acquired solely from the patients themselves. On the day of surgery, PRP processing was performed by qualified personnel: 1 cc of PRP was prepared using the Ycellbio PRP kit (YCBM, Seoul, Korea) from 15 cc of procured blood. To activate PRP without additives (thrombin or CaCl_2_), procured PRP was smoothly perfused between two lure lock syringes.

### Sinus augmentation technique, implant placement, biopsies and prosthetic fabrication

**Fig. 4:**
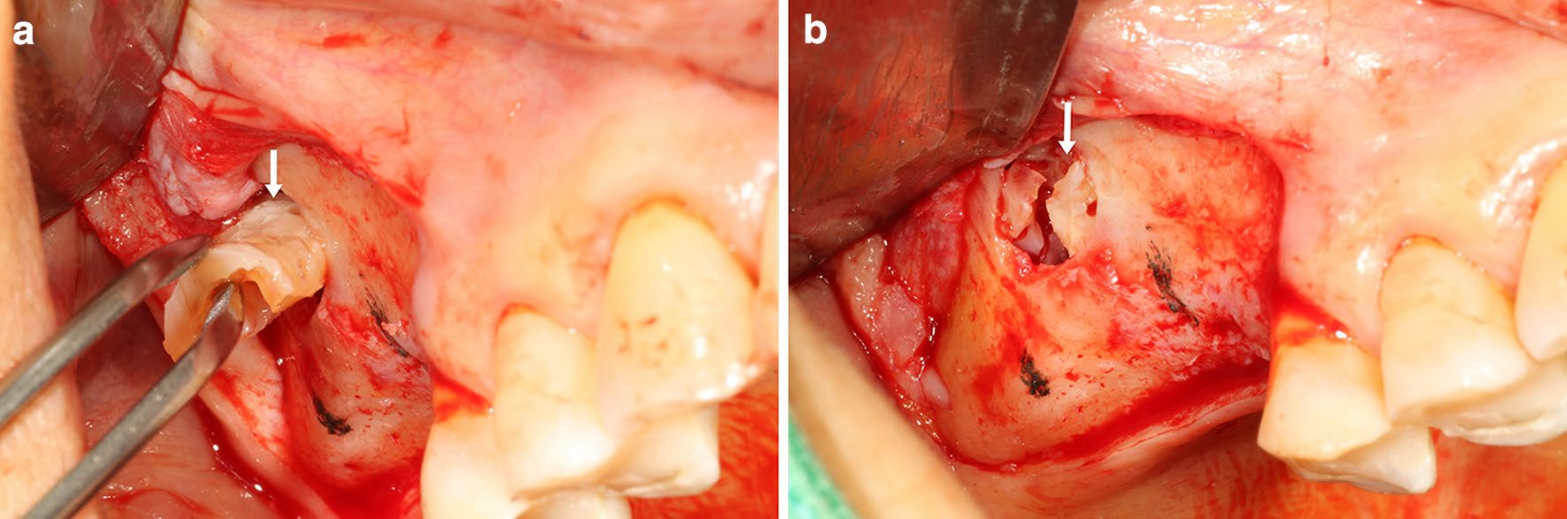
Intraoral photos of sinus graft surgery using Auto-FDT block. **a** The graft before being inserted in the sinus cavity. **b** The completion of the Auto-FDT block grafting. Auto-FDT block (*white arrow*)

Each patient’s mouth was rinsed preoperatively with a 0.1 % chlorhexidine solution (Hexamedine, Bukwang Pharm. Co., Ltd., Seoul, Korea) for 2 min. Surgery was performed under local anesthesia. In the edentulous posterior maxilla, a crestal incision was made slightly palatal with buccal releasing incisions mesially and distally. A small oval-shaped window was prepared in the sinus wall, and the Schneiderian membrane was elevated. In Group 1, the Auto-FDT block bone substitute materials were grafted (Fig. 4a, b). In Group 1, enough material for one molar or two premolars (four pieces) was used. The void space in the Auto-FDT blocks was filled with 1 cc PRP. In the Group 2, a double-layered grafting technique (Lee and Kim 2009) using allograft and bovine xenograft powder (1:1) was applied with 1 cc PRP. The amount used in Group 2 was 1.8 cc.

Dental implants (TSIII CA, Osstem, Seoul, Korea) measuring 4.0–5.0 mm in diameter and 8.5–11.5 mm in length were placed in the edentulous posterior maxilla with simultaneous bone grafts. All implants achieved initial stability with an insertion torque over 10 Ncm. The sutures were removed 10 days after surgery. After augmentation and implant installation, postoperative panoramic radiography was performed to visualize the region.

After a healing period of 6 months, the biopsy was performed in ten patients with additional consent (Group 1, n = 5, Group 2, n = 5). During the uncovering surgery, drilling with the trephine bur (Dentium Co., Suwon, Korea) was performed on the sinus graft site of the alveolar crest. Residual alveolar bone and the whole augmented sinus were included in the biopsies.

Approximately 2–4 weeks after the uncovering surgery, the final restorations were completed. Dental implant stability was measured with a radio frequency device (Osstell Monitor Integration Diagnostics, Goteburg, Sweden) before impressions were taken for the prosthesis fabrication. The radio frequency value was measured twice in two directions (buccal and palatal). The patients were periodically recalled and followed up for 2 years after the prosthetic restoration.

The dental implant success rate was measured on radiographs and clinical evaluations. Implant failure indicates an implant that should be or already has been removed.

### Radiographic evaluations

Before sinus grafting and implant placement, panoramic radiography was performed to evaluate the maxillary sinus state. The residual alveolar height (RAH) and sinus height (SH) were measured using panoramic images immediately after the bone graft and implant installation and 2 year after the final prosthesis fabrication.

**Fig. 5:**
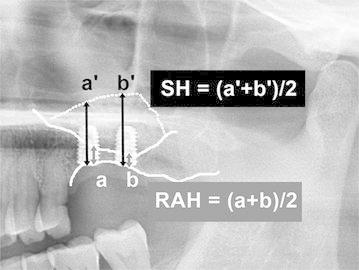
A drawing used to calculate the bone height in a panoramic radiography of the immediate graft. *SH* sinus height, *RAH* residual alveolar height. Heights corresponding to each of the implants from a patient were calculated as the average values

To evaluate the height of the grafted sinus floor, the following variables were used: (1) implant length (IL), which was defined as the distance from the apex to the head of the fixture; (2) residual alveolar height (RAH), which was defined as the distance from the intraoral marginal bone to the lowest point of the original sinus floor in the center of the inserted implant; (3) grafted sinus height (SH), which was defined as the distance from the intraoral marginal bone to the grafted sinus floor directly above the center of the implant (the average of the RAH and SH at the longitudinal center of each implant was measured in one sinus as shown in Fig. 5, and to evaluate the changes in SH after the sinus graft, SH was measured immediately after the operation (SH1) and 2 years after the final fabrication (SH2); (4) to evaluate the augmented graft height (AGH), the difference between the SH1 and RAH was calculated from an immediate post-operative panoramic radiograph (AGH = SH1 − RAH); (5) the resorption height (RH) was defined as the difference between SH1 and SH2 (RH = SH1 − SH2). If the border of the graft material was unclear, then it was not measured.

### Histomorphometry

To assess the new bone formation and histomorphometry, ten biopsies were taken from both groups at 6 months after the sinus augmentation (Group 1, n = 5, Group 2, n = 5). The samples were fixed in 4 % buffered formaldehyde and dehydrated in graded series of alcohols and embedded in paraffin. Four to 6 μm serial longitudinal sections of paraffin-embedded fragments were obtained and stained with hematoxylin-eosin (H&E) and Masson’s trichrome (MT).

For the analyses, a measuring area was determined in the sinus augmentation space above the residual alveolar bone on a low-magnification histological image. The RAH measurements from immediate postoperative panoramic radiography were suitable for detecting residual alveolar bone on low-grade histologic images using an image analysis program. Histomorphometry of the areas of newly formed bone and residual grafted material marrow spaces was performed using a light microscope (Laborlux S; Leitz) connected to a high resolution digital camera (OLYMPUS DP27, Tokyo, Japan) with cellSens (OLYMPUS, Tokyo, Japan) interfaced to a monitor and PC (DM301S1A-AS32, Samsung, Seoul, Korea). Measurement fields were selected by visually monitoring the microscopic screen image (100× magnification). Sections were marked to delineate the margin along the sinus space, including the graft materials and new bone. The new bone and residual graft material volume were presented as the area (mm^2^) and percentage (%).

### Statistical methods

The data were analyzed using a commercial statistical software package (SPSS for windows ver. 20.0). The Significance of between-group differences was assessed with the Mann–Whitney U test. The significant level was set at *P* < 0.05. The height data were classified according to the values measured from the radiographs and treatment records. A statistical program was used to evaluate the resorption height and the differences between the graft materials. The histomorphometry data are presented as the mean and standard deviation. Initially, a mean histomorphometrical value was obtained for each sample.

## Abbreviations

Auto-FDT block: autogenous fresh demineralized tooth block
IL: implant length
PRP: platelet-rich plasma
RAH: residual alveolar height
SH: sinus height
AGH: augmented graft height
RH: resorption height
